# Early non-invasive ventilation for acute respiratory failure in immunocompromised patients (IVNIctus): study protocol for a multicenter randomized controlled trial

**DOI:** 10.1186/1745-6215-15-372

**Published:** 2014-09-25

**Authors:** Virginie Lemiale, Matthieu Resche-Rigon, Elie Azoulay, Study Group for Respiratory Intensive Care in Malignancies (Groupe de Recherche en Réanimation Respiratoire du patient d’Onco-Hématologie, GRRR-OH)

**Affiliations:** Medical ICU, Assistance Publique Hopitaux de Paris, St Louis Hospital, 1 avenue Claude Vellefaux, 75010 Paris, France; Department of Biostatistics, Assistance Publique Hopitaux de Paris, St Louis Hospital, 1 avenue Claude Vellefaux, 75010 Paris, France; Faculté de Medicine, Université Paris-Diderot, Sorbonne Paris-Cité, 5 Rue Thomas Mann, 75013 Paris, France

**Keywords:** Non-invasive ventilation, Immunocompromised patient, Acute respiratory failure

## Abstract

**Background:**

Acute respiratory failure (ARF) remains the leading reason for intensive care unit (ICU) admission of immunocompromised patients. In the most severe cases, high-flow oxygen therapy may fail to ensure adequate gas exchange, and mechanical ventilation (MV) must be used. This scenario is associated with high mortality rates of 40 to 60%, depending on the cause of ARF and type of immune deficiency. The use of non-invasive ventilation (NIV) in this situation has been criticized as potentially delaying the initiation of optimal treatment. In contrast, early NIV used prophylactically in patients with ARF who do not meet the criteria for invasive MV (IMV) may obviate the need for IMV, thereby decreasing the morbidity and mortality rates. We aim to demonstrate that a management strategy including early NIV decreases 28-day mortality rates compared to oxygen therapy alone in immunocompromised patients with ARF.

**Methods/Design:**

This is a multicenter parallel-group randomized controlled trial comparing early NIV to oxygen therapy alone in immunocompromised patients with ARF. All immunocompromised adult patients admitted to admission for ARF are eligible for randomization. Patient with ARF onset more than 72 hours earlier or ARF related to cardiogenic pulmonary edema or hypercapnia, or with a need for immediate endotracheal intubation or other organ failure are not eligible.

After inclusion patient are allocated to receive early NIV (intervention arm) or oxygen therapy only (control arm).

We plan to enroll 374 patients in 29 ICUs. An interim analysis is planned after the inclusion of 187 patients. The main objective is to demonstrate early NIV increases survival as compared to oxygen therapy alone. Other outcomes include the need of IMV, organ failure evolution, nosocomial infections rate, 6 months survival.

**Discussion:**

This study is expected to demonstrate an improved 28-day survival in immunocompromised patients managed with early NIV.

**Trial registration:**

Registration number: Clinicaltrials.gov NCT01915719. Registered on 26 July 2013.

**Electronic supplementary material:**

The online version of this article (doi:10.1186/1745-6215-15-372) contains supplementary material, which is available to authorized users.

## Background

Acute respiratory failure (ARF) in immunocompromised patients is both common and severe [1]. ARF is the leading cause of ICU admission in patients with hematological or solid malignancies. The most recent studies have found mortality rates of nearly 50% in this population [1–3]. Mortality rates are highest in patients with severe respiratory distress requiring invasive mechanical ventilation (IMV). In this situation, mortality can reach 40% in organ transplant recipients and patients receiving immunosuppressive treatments [4], and 60% in patients with hematological or solid malignancies [1–3].

**Figure 1:**
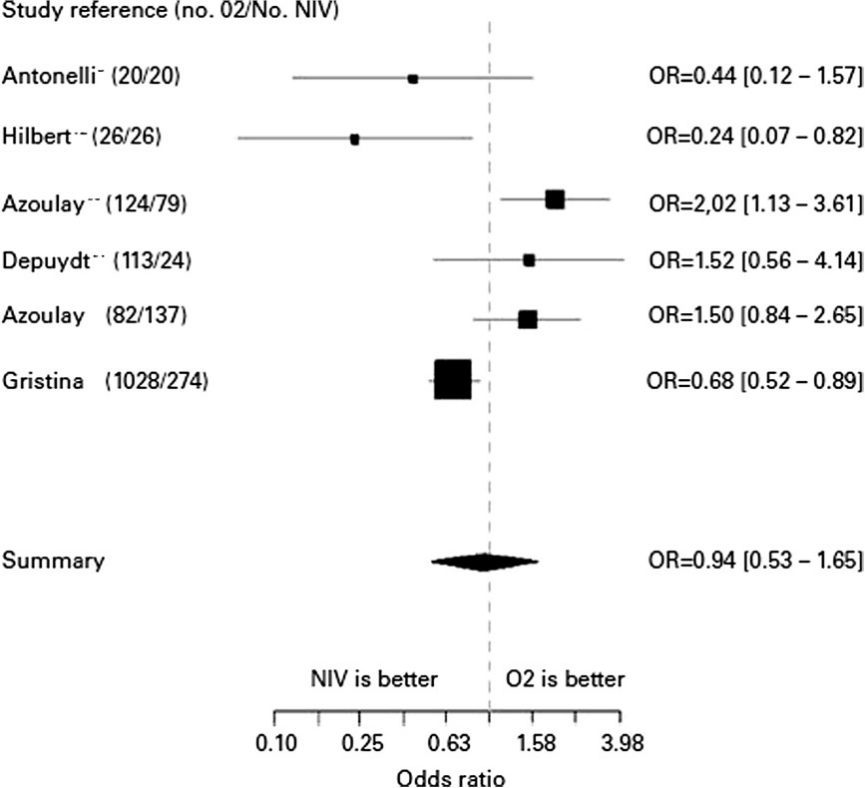
**Outcomes associated with the in-ICU use of non-invasive ventilation in immunocompromised patients.** Adapted from Azoulay E, ^13^.

Noninvasive ventilation (NIV) via a mask was introduced in the 1990s with the primary goal of decreasing the need for IMV and the rate of IMV-related complications. NIV has been chiefly evaluated in two indications: (a) as an alternative to IMV in patients with severe ARF meeting criteria for IMV, and (b) as a means of avoiding IMV in patients with ARF who do not meet criteria for IMV. The use of NIV in patients with severe ARF has been criticized, as the failure rate is nearly 50% among immunocompromised patients and excess mortality occurs among patients who receive delayed IMV [5–7]. Some studies even found improved survival after early IMV [5, 6, 8]. NIV is an accepted treatment when criteria for IMV are not met, although the underlying level of evidence is low [9–12]. A single randomized trial in 52 patients established that early NIV improved survival in immunocompromised patients [11]. However, this study has three major flaws: (a) the patients were recruited at a single center; (b) the mortality rate among patients managed with IMV was 90%, which is considerably higher than current rates; and (c) acute illness severity varied across patients, with some patients apparently meeting criteria for IMV at baseline and others having no criteria for severe ARF. Nevertheless, the beneficial effect of NIV on survival was impressive: mortality was 81% without NIV and 50% with NIV. The result of an Italian study of 40 solid organ transplant recipients supports the beneficial effect of postoperative NIV on survival [12].Figure 1 illustrates these data by showing the results of studies reporting outcomes of immunocompromised patients with ARF. We compared hospital mortality in patients managed with oxygen alone and in patients given NIV.

This forest plot figure represents cohort studies or trials reporting both patients receiving oxygen or NIV. Studies are presented with first author name, year of publication (number of patients treated with oxygen only and number of patients receiving NIV and oxygen). Odd Ratio (OR and [95% CI] comparing mortality associated with the use of NIV.

These studies include: two randomized ICU trials comparing two strategies for managing immunocompromised patients with ARF (Antonelli and Hilbert) [11, 12]; a 2004 prospective nonrandomized trial evaluating the impact of ARF in patients with hematological or solid malignancies (Azoulay) [1]; an ICU study in non-intubated patients with ARF randomized to two different diagnostic strategies (Azoulay) [13]; a retrospective study of ICU patients with ARF managed with oxygen only or NIV (Depuydt) [14]; and a 2011 multicenter Italian observational ICU study of patients with hematological malignancies and ARF (Gristina) [15].

This literature review shows no difference in mortality rates between patients managed with oxygen only and those given NIV. However, none of these studies provides a sufficient level of evidence. In addition, patients in whom ARF was combined with other organ failures were not separated from those with isolated ARF. This is an important point, as the risk of mortality is known to increase with the number of organ failures and the severity of the ARF. Overall, these studies suggest that NIV may be useful as a prophylactic treatment to obviate the need for IMV. In contrast, the use of NIV as an alternative to IMV in patients with severe ARF seems to adversely impact survival.

We have planned a multicenter randomized controlled trial in immunocompromised patients with ARF. This trial will be carried out in 29 specialized ICUs in France and Belgium (in teaching hospitals, community hospitals, and national cancer network hospitals) with high admission rates of immunocompromised patients. The study objective is to demonstrate that incorporating early NIV into the management strategy for patients with ARF who do not meet criteria for IMV improves survival compared to oxygen only.

## Methods/Design

The IVNIctus trial is a prospective, multicenter, open-label, randomized controlled trial comparing a management strategy including early NIV with oxygen alone in immunocompromised patients admitted to ICU with ARF. Early NIV is defined as NIV treatment for patients with ARF who are without any other organ failure or intubation criteria (intubation criteria would be described further (in patient management bias paragrah)).

The study hypothesis is that early NIV decreases mortality in immunocompromised patients requiring ICU admission for ARF. The primary evaluation criterion is survival on day 28 after randomization. Secondary evaluation criteria are proportion of patients treated with IMV in each group, number of organ failures (Sequential Organ Failure Assessment score) 72 hours after randomization in each group, invasive mechanical ventilation duration and ICU stay length in each group, patient comfort in each group, frequency of nosocomial infections in each group, survival at six months after randomization.

The primary study objective is to demonstrate that in immunocompromised patients admitted to the ICU with ARF, a management strategy including early NIV improves 28-day mortality compared to high-flow oxygen without NIV.

### Ethics

The study was approved by the local independent ethic committee (Comite de protection des personnes CPP Ile de France IV, Saint Louis on 21 May 2012, number 2012/11SC), the French health authorities (AFSSAPS) on 11 5 2011, number EudraCT 2011-A00591-40. The University Hospital of Paris (AP-HP) is the sponsor of the trial. Informed consent will be obtained from each participant.

### Eligibility criteria

Only patients meeting all inclusion criteria may be included. Inclusion criteria are as follows: age >18 years; ICU admission; immune deficiency due to any cause (solid organ transplantation, hematological or solid malignancy under treatment or in remission for less than five years, or immunosuppressive treatment defined as corticosteroid therapy (1 mg/Kg/day prednisone equivalent or corticosteroid therapy for longer than one month) or use of another immunosuppressant drug (in a high dosage or for longer than one month); ARF (other than during the postoperative period after cancer surgery) defined as hypoxemia <60 mmHg on room air and/or clinical evidence of respiratory distress (intercostal recession or polypnea >30/min or dyspnea at rest); and onset of ARF within the last 72 hours.

Patients meeting any of the following exclusion criteria will be excluded from the study: hypercapnia >50 mmHg or greater than 10 mmHg PaCO_2_ increase after oxygen therapy initiation, requirement for immediate IMV, probable cardiogenic acute pulmonary edema, requirement for vasoactive therapy (epinephrine or norepinephrine >0.3 μg/Kg/min), impaired consciousness (Glasgow Coma Scale score <13), do-not-intubate decision, long-term oxygen therapy, refusal of the patient or family to provide consent to the study, ongoing myocardial infarction or acute coronary syndrome, pregnancy or breastfeeding or absence of coverage by the French statutory health insurance system.

### Description of the study methodology

#### Study arms

The control group will consist of patients included in the study and allocated at random to the arm managed with oxygen but no NIV. In patients requiring intubation, use of NIV to secure the procedure is allowed. The intervention group will consist of patients included in the study and allocated at random to the arm managed with one hour NIV sessions in addition to oxygen. A total of six hours is required on the first day.

### Patient recruitment modalities

All 29 recruiting ICUs (Additional file 1: Table S1) have considerable experience and expertise in the management of immunocompromised patients. The 29 ICUs participating in the study each admit 50 to 200 immunocompromised patients per year, including the 10 to 20% who meet IVNIctus trial eligibility criteria. Consecutive patients admitted to these ICUs and meeting all the inclusion criteria and none of the exclusion criteria will be included into the trial.

### Randomization and study treatment

Eligible patients will be randomly assigned, in a 1:1 ratio, to one of the two treatment arms. Randomization will occur either at ICU admission or when the eligibility criteria are met if the patient was admitted for a reason other than ARF. Randomization will be performed via the website telemedicine cleanweb.aphp. After having completed the randomization webpage, the investigator will receive the treatment arm designation. Time zero will be defined by randomization time. Randomization will be stratified by center, oxygen flow rate at randomization (>or ≤9 L/min), and reason of immunosuppression (oncologic and hematologic malignancy or immunosuppressed treatment and solid organ transplantation). All the data will be notified in an e-CRF on the website telemedecine cleanweb.aphp.

In the early NIV group (intervention group), NIV sessions will be administered throughout the 24-hour cycle, with oxygen therapy sessions in the intervals, the objective being to maintain SpO_2_ > 92%. NIV would be given for at least six out of 24 hours during the first 24 hours. In the oxygen-only group (control group), continuous oxygen therapy will be provided to maintain SpO_2_ > 92%.

### Treatments and procedures allowed during the trial

#### Bronchoalveolar lavage

BAL may be performed under NIV in both groups if required by the patient’s condition. The efficacy and safety of an NIV session used to perform bronchoscopy have been evaluated previously [16]. NIV duration and parameters will be recorded in the patient’s case report form.

#### Preoxygenation before intubation

This technique consisting in a NIV session immediately before endotracheal intubation has been shown to decrease the decline in oxygen saturation during intubation [17]. It may be used in both groups.

#### Intravenous treatments

Participation in the trial does not result in any restrictions in the use of specific treatments required by the patient’s underlying disease and/or ARF.

### Detailed description of the conduct of the study

**Figure 2:**
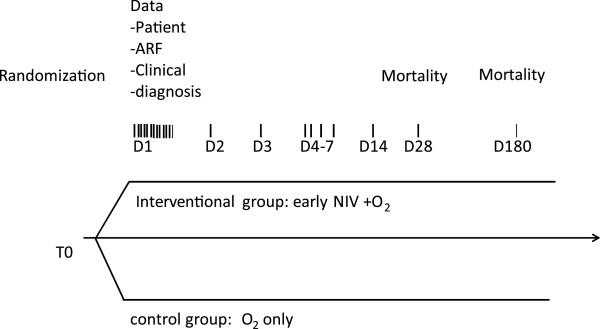
**Study diagram.** ARF, acute respiratory failure; NIV non-invasive ventilation.

Figure 2 shows a diagram of the study procedures.

Table 1 summarizes the clinical and laboratory test performed throughout the study.

**Table 1:** **Clinical and laboratory data**

	Clinical data	Laboratory data
To: randomization	Underlying disease	
	ARF characteristics	
First 24 hours	Respiratory rate, SpO_2_, oxygen flow or FiO_2_	
Day1 to Day7, Day14, Day28	Respiratory data	SOFA score
	Course of respiratory and other organ failure	
	Suspected diagnosis every day	
	Tolerance of NIV in NIV group	
	Intubation criteria in both groups	
Day28	Mortality	
	Diagnosis of ARF	
	Respiratory investigations	
	Hospital acquired infections	
Day180	Mortality	

SpO2: oxygen pulse saturation; FiO2: inspired oxygen fraction; SOFA: Sequential Organ Failure Assessment; ARF: acute respiratory failure; NIV non-invasive ventilation, T0: time of randomization.

### Evaluation at study inclusion (T0)

The evaluation at study inclusion will consist of clinical characteristics of the patient, underlying disease, disease responsible for ARF, and investigations usually performed at ICU admission of immunocompromised patients with ARF.

### Evaluations during study participation

Evaluations performed during study participation will consist of respiratory characteristics (respiratory rate, SpO2, oxygen flow or FiO2) every hour within the first 24 hours; data to assess the disease course on day 1, 2, 3, 4, 5, 6, 7, 14, and 28 (the worst value will be recorded); clinical data; results of investigations; and data on NIV tolerance and efficacy.

### Evaluation at the end of study participation

Evaluations performed at the end of study participation will consist of mortality on day 28, ICU length of stay, hospital length of stay, time on IMV, and mortality on day 180.

### Bias minimization strategies

#### Recruitment bias

Before randomization, no patients will receive humidified high-flow oxygenation. Severity of ARF will be only assessed with the oxygen flow need at randomization, through Venturi mask or nasal cannula. After randomization, patients will receive a humidified high-flow cannula according physician. Humidified high-flow cannula use will be recorded and analyzed.

We will include only patients with ARF but no circulatory failure at baseline (defined by the need of vasopressor over 0.3 μ g/Kg/min), to obtain a population of patients who have severe disease but do not meet the criteria for IMV. We will include consecutive immunocompromised patients, with no restrictions based on type of disease or treatment, in order the replicate usual ICU practice. Patients in whom HIV infection is the only cause of immunodeficiency will not be included since recent data show differences in outcomes between HIV-infected patients and patients with other causes of immune deficiency. HIV-infected patients with other immunocompromised disease will be included.

#### Patient management bias

We will use predefined NIV parameters, oxygen therapy parameters, and IMV criteria to standardize patient management.

### Non-invasive ventilation parameters

We will use the NIV settings recommended for the management of ARF, in pressure support ventilation. The mask most appropriate for the patient will be selected and adjusted to minimize leakage and pressure points. The level of inspiratory assistance will be increased gradually to obtain a tidal volume (V_T_) of 7 to 10 mL/Kg with a respiratory rate (RR) <25/min. The positive expiratory airway pressure will be increased from 2 to 10 cm H2O to obtain a FiO_2_ < 65%. The FiO_2_ value will be set to keep SpO_2_ > 90%. The inspiratory trigger will be set at the lowest possible value. The physician in charge of the patient will check that asynchronous cycles are kept to a minimum (number of asynchronous cycles/total number of cycles <10%). The ventilatory settings will be chosen based on continuous SpO_2_ monitoring and arterial blood gas values. NIV will be used for at least six hours within 24 hours for two days, in sessions of at least 30 to 45 minutes, with oxygen therapy in the intervals.

Criteria for weaning off NIV are as follows: resolution of the signs of respiratory distress, RR <25/min between NIV sessions, P/F >200 for 24 hours, or the patient refuses NIV and does not meet the criteria for IMV. In patients requiring NIV for longer than 12 out of 24 hours or over more than five days, the need for IMV should be evaluated and the level of NIV use considered excessive.

### Oxygen parameters

The oxygen flow rate delivered through a mask will be set to maintain SpO_2_ > 90%. The choice of the oxygen therapy system will be at the discretion of the physician.

For patients receiving oxygen through a mask, FIO2 will be estimated according to the oxygen delivery system and oxygen flow.

### Invasive mechanical ventilation criteria

The criteria for IMV are: severe hemodynamic instability (norepinephrine or epinephrine >0.5 μ g/Kg/min), cardiorespiratory arrest, ongoing myocardial infarction, severe encephalopathy (Glasgow Coma Scale score <11), severe airway secretion retention, worsening of respiratory distress (SpO_2_ < 92% or RR >40/min) regardless of the oxygen flow rate or use of accessory muscles of respiration, inability to maintain PaO_2_ > 65 mmHg with FiO_2_ > 0.6, dependency on NIV with inability to remain off NIV for longer than two hours, or >50% increase in the time on NIV from one day to the next (for example, six hours of NIV on D1 followed by more than nine hours on D2).

### End of the trial

Each patient will be in the trial for six months. Premature study withdrawal will be considered if one of the following occurs: death within the first 28 days, or if requested by the patient or next of kin. Patients withdrawn prematurely from the trial should undergo the procedures scheduled for the last trial visit, to the extent possible. Furthermore, the reason for premature trial withdrawal must be recorded both in the case-report form and in the source document.

### Statistical analysis

The analysis will be performed according to the intention-to-treat approach. We will use the chi-square test to compare 28-day mortality rates in the group managed with oxygen only and in the group managed with oxygen and early NIV. Randomization will be stratified on the underlying disease (hematological or solid malignancy versus other) and on ARF severity as assessed by the baseline oxygen flow rate (≤9 L /min versus >9 L/min to obtain SpO_2_ > 95% and/or resolution of the signs of respiratory distress). We will test for an interaction between the treatment effect and the underlying disease. If such an interaction is found, we will use the chi-square test to look for a treatment effect within the stratification subgroups. We will correct for the multiple comparisons using the Bonferroni test (error of first order 0.025). Similarly, we will look for an interaction between the treatment effect and baseline ARF severity. If such an interaction is found, we will use the chi-square test to look for a treatment effect within the stratification subgroups. We will correct for the multiple comparisons using the Bonferroni test (error of first order 0.025).

For the secondary evaluation criteria, we will use the chi-square test to compare the proportions of patients with treatment failure (defined by the need of IMV), and nosocomial infection in the two treatment arms. The procedure described above for potential subgroup analyses of the primary evaluation criterion will be used for these secondary evaluation criteria. Wilcoxon’s test will be performed to compare the number of organ failures (SOFA score) between the two arms 72 hours after study inclusion. Median hospital and ICU length of stay will be compared between the two arms using the Kaplan-Meier estimator, with discharge alive as the event of interest and death as the censoring event. Time on invasive mechanical ventilation will be compared between the two arms using the log-rank test.

### Sample size estimation

Based on previous studies in the field and on evidence of declining mortality rates over the last decade among ICU patients with hematological or solid malignancies [12, 13, 18, 19], we expect 28-day mortality rates of 20% in the early NIV arm and 35% in the oxygen-only arm. With alpha set at 0.05, 90% power, and the chi-square test, 187 patients are needed in each arm, totalling 374 patients in all. A single interim analysis to assess superiority is scheduled. The interim analysis will take place after inclusion of half the patients (n = 187). The criteria for premature study discontinuation based on the results of this single interim analysis will be those defined by O’Brien and Fleming [20]. All tests will be two-sided. The statistical analysis will be performed using SAS 9.1.3 (SAS, Inc., Cary, NC, USA) (or a more recent version) or R.2.15.2 (R foundation for statistical computing, Vienna, Austria). (or a more recent version).

### Provisional time table

Based on the recruitment rates in the TRIAL-OH study [21] of consecutive hematology patients admitted to the same ICUs (appendix 4, study ongoing), the 30% proportion of patients with ARF who were not intubated at ICU admission in the same study, and the admission rate of immunocompromised patients in the study centers, recruitment is expected to last approximately 30 months. The Data and Safety Monitoring Committee will meet regularly to review adverse events recorded during the trial. The overall data analysis will be performed under the responsibility of Professor Chevret and Dr Resche-Rigon at the Biostatistics Department of the Saint-Louis Teaching Hospital, Paris, France.

### Data and Safety Monitoring Committee

An independent data and safety monitoring committee will be established. This committee will have three members: Professor S Jaber (Saint-Éloi teaching hospital, Montpellier, France)), Dr CE Luyt (Pitié Salpêtrière, *teatching hospital* Paris *France*), and Professor B Maitre (H*enri* Mondor *teatching hospital*, Crétei*, France*l).

The mission of the Data and Safety Monitoring Committee consists of monitoring the safety data and providing the study sponsor with recommendations about whether the trial should continue as planned, prolonged, or stopped prematurely. It will also be responsible for evaluating the results of the single interim analysis of superiority scheduled to occur after the inclusion of half of the patients (N = 187). The criteria defined by O’Brien and Fleming [20] will be used to determine whether the results of this interim analysis warrant premature discontinuation of the trial.

## Discussion

ARF remains the most frequent and challenging life-threatening event in patients with hematological malignancies [1, 5–7, 14, 18]. In patients with prolonged neutropenia (acute leukemia or *bone marrow tranplant* recipients), respiratory events occur in up to half of cases, of which a further half are complicated by ARF [22]. Despite a recent improvement in survival, IMV remains associated with high mortality in immunocompromised patients with ARF [21–24]. NIV has been associated with an increase survival for these patients, in an era when IMV was associated with a mortality rate of over 80% [9, 11]. In recent studies, mortality after intubation was 60% in hematological patients and 40% in immunocompromised patients [5, 13, 19, 21]. In that setting, appropriate management of the patients with ARF became uncertain.

Moreover, a crucial point is the difference between early and late NIV. Early NIV is used in patients who do not meet criteria for IMV. In contrast, late NIV is given as an alternative to IMV. Several recent studies found higher mortality rates among patients who failed NIV than among patients managed with first-line IMV [5, 19]. In addition, in recent years outcomes have improved among immunocompromised patients admitted to the ICU, including those managed with IMV [5, 7, 19, 21]. This improvement is ascribable not only to better patient selection for ICU admission, but also to better overall management of the underlying disease and ARF. Most of the studies evaluated NIV without really controlling time between ARF onset to NIV implementation, without taking into account ARF etiology, and/or without taking into account the presence of associated organ dysfunction at the time of NIV initiation. As a consequence, prophylactic NIV (in patients with hypoxemia but no respiratory distress) and curative (patients with respiratory distress needing ventilatory support) were lumped together.

We expect this trial to assess a management strategy including early NIV and to identify risk factors for IMV among immunocompromised patients with ARF.

## Trial status

The trail is currently recruiting patients. Inclusion started on 1 September 2013 and the number of included patients so far is 162. The estimated length of inclusion time is 30 months.

## Electronic supplementary material

Additional file 1: Table S1: List of investigators involved in the study. (DOCX 15 KB)

## Abbreviations

ARF: Acute respiratory failure
ICU: Intensive care unit
IMV: Invasive mechanical ventilation NIV, Non-invasive mechanical ventilation.
