# Supplementary material for: Early non-invasive ventilation for acute respiratory failure in immunocompromised patients (IVNIctus): study protocol for a multicenter randomized controlled trial
Source: Trials. 2014 Sep 25;15:372. doi: 10.1186/1745-6215-15-372 (PMC4190291; doi:10.1186/1745-6215-15-372)
Supplement: Supplementary file 1 — Additional file 1: Table S1: List of investigators involved in the study. (DOCX 15 KB) [file 13063_2014_2243_MOESM1_ESM.docx]

| Centre N° | Investigator | Address |
| --- | --- | --- |
| 1 | Dr Lemiale | Medical ICU, Saint Louis Hospital, Paris |
| 2 | Dr Pène | Medical ICU, Cochin Hospital, Paris |
| 3 | Dr Ekpe | Gustave Roussy Institute, Villejuif, France |
| 4 | Pr Benoit | ICU, University Hospital Ghent, Belgium, France |
| 5 | Dr Mayaux | Medical ICU, Pitié-Salpétrière Paris, France |
| 6 | Pr Darmon | ICU, University Hospital,Saint Etienne, France |
| 7 | Dr Chemouni | Medical ICU, Avicenne Hospital, Bobigny, France |
| 8 | Dr Mokart | Paoli Calmettes Institute, Marseilles, France |
| 9 | Dr Kouatchet | ICU, University Hospital Angers, France |
| 10 | Dr Bruneel | ICU, Mignot Hospital, Versailles, France |
| 11 | Dr Perez | UCI University Hospital Nancy, France |
| 12 | Dr Hamidfar-Roy | ICU, University Hospital Grenoble, France |
| 13 | Dr N’Yunga | ICU Roubaix Hospital, France |
| 14 | Dr Meert | J Bordet Institute, Bruxelles, Belgium |
| 15 | Dr Reigner | ICU La Roche sur Yon Hospital, France |
| 16 | Dr Renaud | ICU University Hospital Brest, France |
| 17 | Dr Mariotte | Medical ICU, Bichat Hopital, Paris, France |
| 18 | Dr Feisel | ICU Belfort Hospital, France |
| 19 | Dr Guitton | ICU, University Hospital Nantes, France |
| 20 | Dr Barbier | ICU University Hospital Orléans, France |
| 21 | Pr Argaud | Medical ICU, Herriot Hospital, Lyon, France |
| 22 | Dr Seguin | Medical ICU, University Hospital,Caen, France |
| 23 | Pr Papazian | Medical ICU, University Hospital, Marseille, France |
| 24 | Dr Schenck | Medical ICU, University Hospital Strasbourg, France |
| 25 | Dr Rabbat | Pulmonary ICU, Cochin Hospital, Paris, France |
| 26 | Dr Moreau | Medical ICU, University Hospital, Lille, France |
| 27 | Dr Loay | ICU Amiens Hospital, France |
| 28 | Dr Girault | Medical ICU, University Hospital, Rouen, France |
| 29 | Dr Choukroun | ICU, Sud –Francilien Hospital, Corbeil, France |

Additional file 1: Table S1

Authors’information : participating center
